# Multicenter randomized phase II trial of prophylactic right‐half dissection of superior mesenteric artery nerve plexus in pancreatoduodenectomy for pancreatic head cancer

**DOI:** 10.1002/ags3.12399

**Published:** 2020-09-15

**Authors:** Suguru Yamada, Sohei Satoi, Hideki Takami, Tomohisa Yamamoto, Isaku Yoshioka, Fuminori Sonohara, So Yamaki, Kazuto Shibuya, Masamichi Hayashi, Daisuke Hashimoto, Masahiko Ando, Kenta Murotani, Mitsugu Sekimoto, Yasuhiro Kodera, Tsutomu Fujii

**Affiliations:** ^1^ Department of Gastroenterological Surgery Nagoya University Graduate School of Medicine Nagoya Japan; ^2^ Department of Surgery Kansai Medical University Moriguchi Japan; ^3^ Department of Surgery and Science Faculty of Medicine Academic Assembly University of Toyama Toyama Japan; ^4^ Center for Advanced Medicine and Clinical Research Nagoya University Hospital Nagoya Japan; ^5^ Graduate School of Medicine Biostatistics Center Kurume University Kurume Japan

**Keywords:** pancreatic head cancer, pancreatoduodenectomy, phase II trial, superior mesenteric artery nerve plexus

## Abstract

**Aim:**

Right‐half dissection of the superior mesenteric artery (SMA) nerve plexus in pancreatoduodenectomy for pancreatic cancer was initiated to accomplish R0 resection; however, subsequent refractory diarrhea was a major concern. This study aimed to evaluate the necessity of this technique.

**Methods:**

From April 2014 to June 2018, 74 patients with pancreatic head cancer were randomly allocated to either Group A, in which right‐half dissection of the SMA nerve plexus was performed (n = 37), or Group B, in which total preservation of the nerve plexus was performed (n = 37). Short‐term, long‐term, and survival outcomes were prospectively compared between the groups.

**Results:**

The patient demographics, including the R0 resection rate, were not significantly different between the groups. Postoperative diarrhea occurred in 26 (70.3%) patients in Group A and 18 (48.6%) patients in Group B. There was a tendency for the development of severe diarrhea in Group A within 1 year postoperatively, and the frequency of diarrhea gradually decreased within 2 years, although that did not affect tolerance to adjuvant chemotherapy. There was no difference in either locoregional recurrence (27.0% vs 32.4%) or systemic recurrence (46.0% vs 46.0%). The median overall survival time in Groups A and B was 37.9 and 34.6 months, respectively (*P* = 0.77).

**Conclusion:**

We did not demonstrate a clinical impact of right‐half dissection of the SMA nerve plexus on locoregional recurrence or survival. Therefore, the prophylactic dissection of the SMA nerve plexus is unnecessary given that refractory diarrhea could be induced by this technique (UMIN000012241).

## Graphical abstract

The necessity of right‐half dissection of the SMA nerve plexus in pancreatoduodenectomy for pancreatic cancer was evaluated. We did not demonstrate the clinical impact of this technique on locoregional recurrence or survival. Therefore, the prophylactic dissection is unnecessary, given that refractory diarrhea could be induced by this technique.

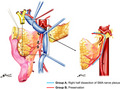

## 1 INTRODUCTION

Pancreatic ductal adenocarcinoma (PDAC) is one of the most lethal malignancies worldwide. It currently ranks as the third leading cause of cancer‐related death and is estimated to become the second leading cause by 2030.^1^ The survival outcome of PDAC is still dismal, with actual 5‐year survival rates ranging from 10% to 20%.^2^ To conquer this disease, extended lymphadenectomy with dissection of the nerve plexus has been challenged in some randomized controlled trials; however, survival outcomes have not been improved.^3^, ^4^, ^5^, ^6^

Among some extended surgery techniques for PDAC, right‐half dissection of the superior mesenteric artery (SMA) nerve plexus has been considered a standard technique of pancreatoduodenectomy in Japan.^7^, ^8^ Although this concept was initiated to accomplish R0 resection and totally extirpate the tissue that had the potential for nerve plexus invasion, subsequent refractory diarrhea is a major concern. This refractory diarrhea often decreases the relative dose intensity of adjuvant chemotherapy, which has been demonstrated to be a significant prognostic factor in PDAC.^9^, ^10^ Therefore, unnecessary right‐half dissection of the SMA nerve plexus in pancreatoduodenectomy might induce refractory diarrhea, potentially worsening the prognosis.

This multicenter randomized phase II trial was performed to judge the necessity of right‐half dissection of the SMA nerve plexus in the context of the locoregional recurrence rate. We randomly assigned the enrolled patients into those who did and did not undergo right‐half dissection of the SMA nerve plexus and statistically analyzed the short‐term, long‐term, and survival outcomes between the groups.

## 2 METHODS

### 2.1 Patient recruitment

From April 2014 to June 2018, 80 patients from Japanese regional high‐volume centers (Nagoya University Hospital, Kansai Medical University Hospital, and Toyama University Hospital) were enrolled in this phase II study. The eligibility criteria were planned pancreatoduodenectomy for pancreatic head cancer, no invasion of the SMA nerve plexus on preoperative imaging studies and estimated R0 resection by preservation of the nerve plexus, and age of ≥20 to ≤75 years. The exclusion criteria were a malignant pancreatic tumor other than pancreatic cancer, invasion or suspected invasion of the SMA nerve plexus, liver cirrhosis or active hepatitis, interstitial pneumonia or emphysema, artificial dialysis for chronic renal failure, other active concomitant malignancies, and preoperative moderate diarrhea. Written informed consent was obtained from all patients. This study was conducted in accordance with the Declaration of Helsinki, and the study protocol was approved by the institutional review board of the affiliated hospital. The registration number of this clinical trial is UMIN000012241.

### 2.2 Study design

**FIGURE 1:**
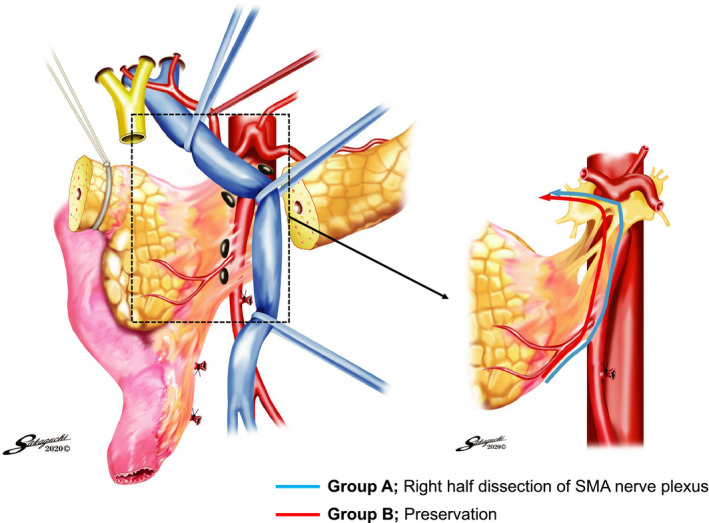
Study schema. The blue line indicates right‐half dissection, and the red line indicates preservation of the SMA nerve plexus. SMA, superior mesenteric artery

**FIGURE 2:**
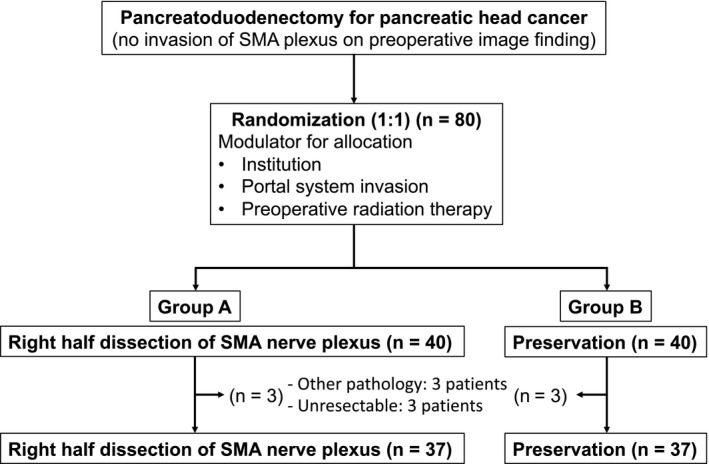
CONSORT diagram. SMA, superior mesenteric artery

In this multicenter randomized phase II study, 80 enrolled patients were randomly allocated to Group A, in which right‐half dissection of the SMA nerve plexus was performed (n = 40), and Group B, in which total preservation of the nerve plexus was performed (n = 40). The modulators of allocation were the institution, portal system invasion, and preoperative radiation therapy. In Group A, the nerve plexus was dissected from the bifurcation of the middle colic artery to the root of the SMA, along with the pancreatic head.^8^ The remnant SMA nerve plexus that was the nearest to the presenting part of the tumor was pathologically evaluated intraoperatively in at least two sites.^11^ In Group B, the pancreatoduodenectomy was performed with exposure and preservation of the nerve plexus in its entire circumference (Figure 1). D2 lymph node dissection was conducted in both groups; thus, the only difference was the performance of right‐half nerve plexus dissection. A close‐up photograph of the SMA was taken to confirm the accuracy of the procedure (Figure S1). The CONSORT diagram of this study is shown in Figure 2. Other pathologies were diagnosed in three patients, and another three patients’ conditions were deemed unresectable; therefore, these six patients were excluded. Finally, 74 patients (37 in each group) were analyzed. The primary endpoint was the local recurrence rate at 2 years after surgery. The secondary endpoints were the frequency of diarrhea, accumulation of ascites, peritoneal recurrence rate, initiation ratio and start of adjuvant chemotherapy after surgery, recurrence‐free survival (RFS), and overall survival (OS). In the current study, zero mm rule was utilized to evaluate the residual tumor (R) status.

### 2.3 Postoperative follow‐up

Oral S‐1 (oral 5‐fluorouracil prodrug tegafur with oteracil and gimeracil) was initiated as adjuvant chemotherapy from 3 to 10 weeks postoperatively. S‐1 was administered from days 1 to 28 followed by a 2‐week rest period.^10^ In outpatient clinics, the patients were followed up once per month for 6 months and every 3 months thereafter. Blood examinations, including measurement of serum tumor markers, were performed at every outpatient care visit, and dynamic computed tomography or magnetic resonance imaging was performed every 3 months.

### 2.4 Statistical analysis

The sample size was calculated as follows. The local recurrence rate at 2 years after surgery in Group A was estimated to be 15% to 20%. Assuming a null hypothesis of 30% and an alternative hypothesis of 10% with a one‐sided type I error of 0.05 and power of 0.9, enrollment of 37 patients per arm was required. Continuous variables are expressed as mean ± standard deviation. RFS was defined as the time from surgery to confirmation of any recurrence. OS was defined as the time from surgery to death by any cause. Survival analysis based on the Kaplan‐Meier method and log‐rank tests were also performed. The last follow‐up date was April 2020. All statistical analyses were performed using JMP Pro version 14.2.0 (SAS Institute, Cary, NC, USA) and R version 3.5.3 (http//www.r‐project.org/). The level of statistical significance was set at *P* < 0.05.

## 3 RESULTS

### 3.1 Patient demographics

Patient demographics are compared between the two study groups in Table 1. There was no difference in age, sex distribution, performance status, or body mass index. Twenty‐nine (78.4%) resectable cancers based on the NCCN guidelines Version 1.2014 were observed in Group A and 30 (81.1%) were observed in Group B, with no difference in the resectability status between the groups.^12^ Neoadjuvant therapy was performed in 16 (43.2%) and 12 (32.4%) patients, among whom preoperative radiation therapy was conducted in only two (5.4%) in Group A and four (10.8%) in Group B, respectively. Among the operative factors, there was no difference in the operation time or blood loss. Pathologically, invasion of the SMA nerve plexus was reported in two (5.4%) and three (8.1%) patients in Groups A and B, respectively, and the R0 resection rate was also comparable between the two groups. As for detailed site of R1, no patient with positive SMA margin was observed in Group A, whereas one was observed in Group B.

**Table 1:** Comparison of demographics between patients with and without right‐half dissection of SMA nerve plexus

	Dissection (n = 37)	Preservation (n = 37)	*P*
*Clinical variables*			
Age, years	64.8 ± 8.9	65.3 ± 9.1	0.8028
Sex, male/female	24 (64.9%) /13 (35.1%)	23 (62.2%)/ 14 (37.8%)	1.0000
Performance status, 0/1	36 (97.3%)/ 1 (2.7%)	36 (97.3%)/ 1 (2.7%)	1.0000
Body mass index, kg/m^2^	21.1 ± 2.8	21.4 ± 2.4	0.5954
NCCN resectability status			
R	29 (78.4%)	30 (81.1%)	0.7105
BR‐PV	7 (18.9%)	5 (13.5%)	
BR‐A	1 (2.7%)	2 (5.4%)	
Tumor size, mm	23.0 ± 7.2	23.8 ± 8.1	0.6513
Biliary drainage	17 (46.0%)	23 (62.2%)	0.2433
Neoadjuvant therapy	16 (43.2%)	12 (32.4%)	0.4725
Preoperative radiation therapy	2 (5.4%)	4 (10.8%)	0.6741
CEA, ng/ml	27.6 ± 126.0	5.2 ± 5.8	0.2865
CA19‐9, U/ml	320.5 ± 827.5	765.2 ± 1713.9	0.1612
DUPAN‐2, U/ml	305.0 ± 415.8	282.8 ± 600.3	0.8756
Operation time, min	450.3 ± 111.7	491.4 ± 108.8	0.1134
Blood loss, ml	892.4 ± 565.8	1024.7 ± 649.1	0.3530
Portal vein resection	16 (43.2%)	21 (56.8%)	0.3525
Blood transfusion	5 (13.5%)	5 (13.5%)	1.0000
Pathological invasion of SMA nerve plexus	2 (5.4%)	3 (8.1%)	1.0000
Residual tumor, R0/R1	35 (94.6%)/ 2 (5.4%)`	31 (83.8%)/ 6 (16.2%)	0.2611
SMA margin	0 (0.0%)	1 (2.7%)	
Dissected pancreatic margin	2 (5.4%)	5 (13.5%)	
UICC stage (7th edition)			
IA	3 (8.1%)	3 (8.1%)	0.9188
IB	1 (2.7%)	0 (0.0%)	
IIA	9 (24.3%)	7 (18.9%)	
IIB	21 (56.8%)	24 (64.9%)	
III	1 (2.7%)	1 (2.7%)	
IV	2 (5.4%)	2 (5.4%)	

Note

Data are expressed as mean ± standard deviation or n (%).

Abbreviations: BR‐A, borderline resectable with arterial invasion; BR‐PV, borderline resectable with portal vein invasion; CA19‐9, cancer antigen 19‐9; CEA, carcinoembryonic antigen; R, resectable; SMA, superior mesenteric artery; UICC, Union for International Cancer Control.

### 3.2 Short‐term outcomes

**FIGURE 3:**
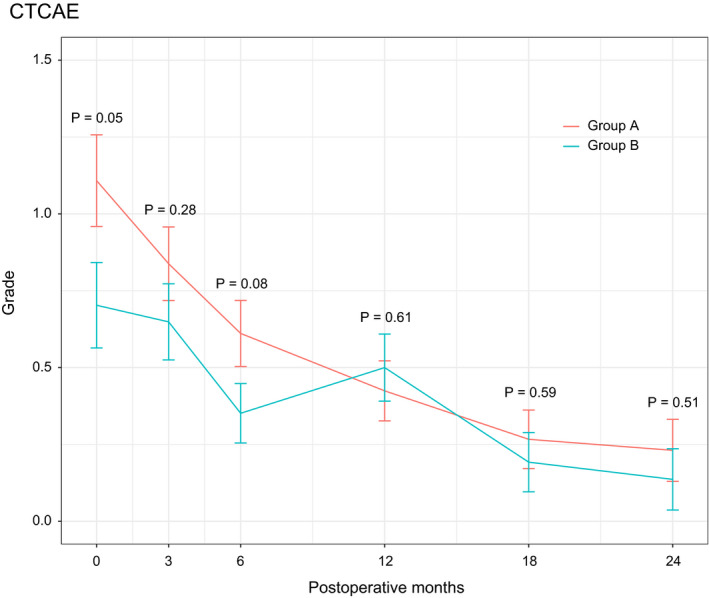
Postoperative course of diarrhea between the two groups. Group A, right‐half dissection of the superior mesenteric artery nerve plexus; Group B, preservation of the nerve plexus

The short‐term outcomes after pancreatectomy are compared between the two study groups in Table 2. Postoperative diarrhea was evaluated based on CTCAE v4.0. A total of 26 (70.3%) patients in Group A and 18 (48.6%) patients in Group B developed postoperative diarrhea. Figure 3 shows the postoperative course of diarrhea between the two groups. There was a greater tendency for the development of severe diarrhea in Group A than B within 1 year postoperatively, and the frequency of diarrhea gradually decreased within 2 years in both groups. Also, the postoperative time‐course alteration of the incidence for clinically significant grade 2 or 3 diarrhea in each group was depicted in Figure S2, which showed the same pattern. Opioid use was found in four patients for Group A and two in Group B. With respect to other complications, we found no difference in the development of pancreatic fistula, ascites, intra‐abdominal infection, delayed gastric emptying, or intra‐abdominal bleeding. No reoperations or 30‐day mortality occurred in this trial. When the status of adjuvant chemotherapy was compared, we found no differences in the initiation ratio, start of treatment after surgery, or completion ratio between the groups.

**Table 2:** Comparison of short‐term outcomes between patients with and without right‐half dissection of SMA nerve plexus

	Dissection (n = 37)	Preservation (n = 37)	*P*
*Complications*			
Diarrhea (CTCAE v4.0)			
Absent	11 (29.7%)	19 (51.4%)	0.2505
Present	26 (70.3%)	18 (48.6%)	
Grade 1	13 (35.1%)	11(29.7%)	
Grade 2	11 (29.7%)	6(16.2%)	
Grade 3	2 (5.4%)	1(2.7%)	
Opioid use as antidiarrheal agent			
Yes	4 (10.8%)	2 (5.4%)	0.6741
Pancreatic fistula^a^			
None	30 (81.1%)	29 (78.4%)	0.6784
Biochemical leakage	3 (8.1%)	5 (13.5%)	
Grade B	3 (8.1%)	3 (8.1%)	
Grade C	1 (2.7%)	0 (0.0%)	
Ascites	13 (35.1%)	12 (32.4%)	1.0000
Intra‐abdominal infection	4 (10.8%)	4 (10.8%)	1.0000
Delayed gastric emptying	4 (10.8%)	4 (10.8%)	1.0000
Intra‐abdominal bleeding	1 (2.7%)	0 (0.0%)	1.0000
Others	9 (24.3%)	7 (18.9%)	0.7784
Re‐operation	0 (0.0%)	0 (0.0%)	1.0000
Postoperative hospital stay, days	18.7 ± 9.0	20.3 ± 13.8	0.5644
30‐day mortality	0 (0.0%)	0 (0.0%)	1.0000
30‐day re‐admission	5 (13.5%)	2 (5.4%)	0.4297
Adjuvant chemotherapy			
Initiation ratio	35 (94.6%)	33 (89.2%)	0.6741
Start of treatment after surgery, days	40.6 ± 15.9	45.6 ± 23.2	0.3093
Completion ratio	29 (82.9%)	21 (63.6%)	0.1003
Performance status, 0/1/2	27 (75.0%)/ 8 (22.2%)/ 1 (2.8%)	28 (82.4%)/ 6 (17.7%)/ 0 (0.0%)	0.5359

Note

Data are expressed as mean ± standard deviation or n (%).

Abbreviations: CTCAE v4.0, Common Terminology Criteria for Adverse Events version 4.0; SMA, superior mesenteric artery.

^a^ Pancreatic fistula was defined based on the 2016 ISGPS definition.

### 3.3 Long‐term outcomes

The long‐term outcomes are compared between the two study groups in Table 3. Recurrence in all sites was observed in 21 (56.8%) patients in Group A and 22 (59.5%) patients in Group B, with no significant difference. When the recurrence rate was evaluated based on the initial recurrence patterns, there was no difference in either the locoregional or systemic (peritoneum, liver, lymph node, and lung) recurrence rates. The confirmed locoregional recurrence rate in Group A was 27.0% (90% confidence interval = 16.9%‐40.3%), whereas, that in Group B was 32.4% (90% confidence interval = 21.4%‐45.9%), so the null hypothesis for the primary endpoint (≤30%) was not rejected.

**Table 3:** Comparison of long‐term outcomes between patients with and without right‐half dissection of SMA nerve plexus

	Dissection (n = 37)	Preservation (n = 37)	*P*
All recurrence	21 (56.8%, 40.9%‐71.3%^a^)	22 (59.5%, 46.1%‐75.9%^a^)	1.0000
Locoregional	10 (27.0%, 16.9%‐40.3%^b^)	12 (32.4%, 21.4%‐45.9%^b^)	0.7997
Systemic	17 (46.0%, 31.0%‐61.6%^a^)	17 (46.0%, 33.4%‐64.1%^a^)	1.0000
Peritoneum	7 (18.9%, 9.5%‐34.2%^a^)	9 (24.3%, 15.4%‐43.0%^a^)	0.7784
Liver	9 (24.3%, 13.4%‐40.1%^a^)	14 (37.8%, 24.1%‐53.9%^a^)	0.3151
Lymph node	5 (13.5%, 5.9%‐28.0%^a^)	3 (8.1%, 2.8%‐21.3%^a^)	0.7106
Lung	6 (16.2%, 7.7%‐31.1%^a^)	2 (5.4%, 1.5%‐17.7%^a^)	0.2611

Note

Data are expressed as n (%).

Abbreviation: SMA, superior mesenteric artery.

^a^ 95% confidence interval.

^b^ 90% confidence interval.

### 3.4 Survival outcomes

**FIGURE 4:**
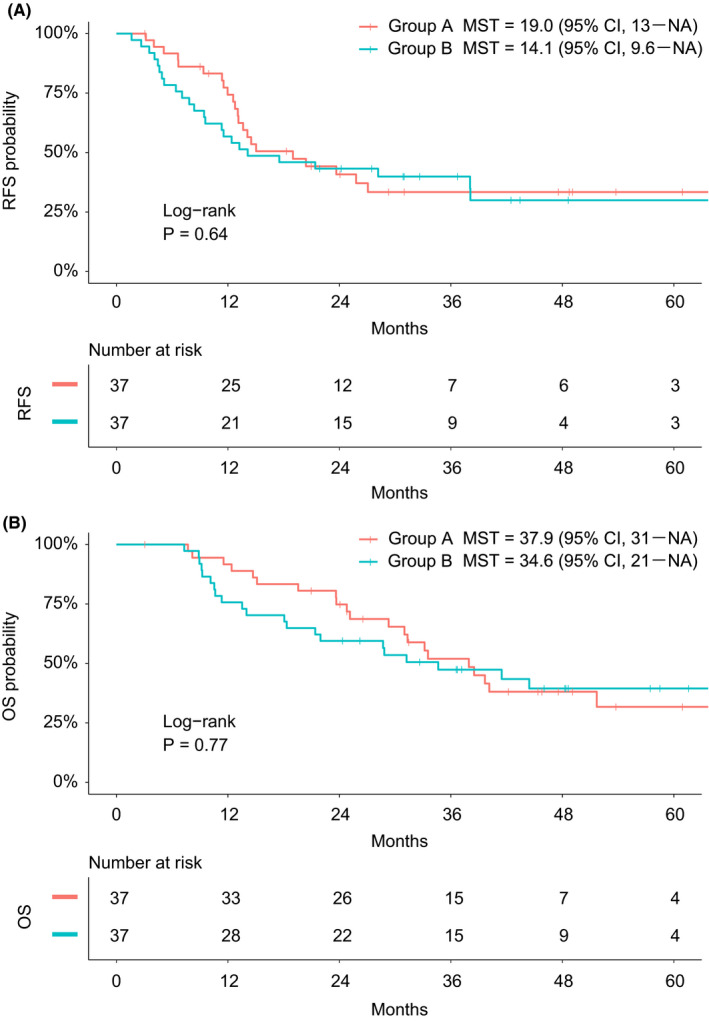
A, Recurrence‐free survival and B, overall survival were compared between the groups. Group A, right‐half dissection of the superior mesenteric artery nerve plexus; Group B, preservation of the nerve plexus. MST, median survival time; CI, confidence interval; NA, not available; RFS, recurrence‐free survival; OS, overall survival

Finally, RFS and OS are compared between the two study groups in Figure 4. The median RFS time in Group A was 19.0 months and that in Group B was 14.1 months, with no significant difference (2‐year OS, 42.4% vs 46.0%; *P* = 0.64). Similarly, the median OS time in Group A was 37.9 months and that in Group B was 34.6 months, which were also comparable (2‐year OS, 74.8% vs 59.5%; *P* = 0.77).

## 4 DISCUSSION

In this trial, we randomized the enrolled patients into Group A, in which right‐half dissection of the SMA nerve plexus was performed, and Group B, in which the SMA nerve plexus was preserved. We then compared the clinical and survival outcomes between the groups. We found no significant difference in either the total recurrence rate or the locoregional recurrence rate between the groups. There was no clinical benefit with respect to long‐term survival in Group A. Rather, nerve plexus dissection induced postoperative diarrhea as expected, which might have a detrimental impact on postoperative adjuvant chemotherapy.

Fortner^13^ first proposed the concept of extended pancreatectomy in the field of pancreatic cancer surgery. In Japan, Ishikawa et al^14^ subsequently advocated that extensive surgery including complete dissection of the lymphatic and connective tissues around the common hepatic artery and SMA could provide a survival benefit compared with conventional surgery. However, later randomized controlled trials did not successfully demonstrate the clinical significance of this extended pancreatectomy.^3^, ^4^, ^5^, ^6^ With respect to dissection of the SMA nerve plexus, some previous reports revealed that perineural invasion around the SMA may be a prognostic factor in PDAC.^15^, ^16^, ^17^ However, we found no randomized controlled trials that reached this conclusion. In 2014, Jang et al^18^ reported that extended lymphadenectomy with dissection of the nerve plexus did not provide a significant survival benefit compared with standard resection in pancreatic head cancer; however, they did not genuinely evaluate the benefit of dissection of the SMA nerve plexus.

Generally, refractory postoperative diarrhea is a major concern after dissection of the SMA nerve plexus. Inoue et al^19^ performed a unique tailored mesopancreas dissection technique and observed postoperative diarrhea in 65% of patients who underwent hemicircumferential nerve plexus dissection and 50% of patients who underwent nerve plexus preservation. In contrast, Jang et al^18^ showed a comparable and relatively low rate of diarrhea between the standard and extended groups (12.0% vs 15.1%, respectively), indicating little effect on intestinal motility. The current study showed a high rate similar to that reported by Inoue et al,^19^ but both studies revealed that most cases of diarrhea were well‐controlled by antidiarrheal opioids. Additionally, we found no difference in the impact on adjuvant chemotherapy between the two groups.

When considering the survival impact of nerve plexus dissection, we must refer to previous randomized controlled trials. However, these studies were conducted a generation ago,^3^, ^4^, ^5^ when eminent procedures such as neoadjuvant and adjuvant chemotherapy were not as available as they are today. Even among studies published in the 2010s, the 2‐year overall survival rate was <50%.^6^, ^18^ In contrast, in the present study, the 2‐year OS rate in the dissection group was 74.8% and that in the preservation group was 59.5%. Thus, comparison with previous studies is difficult. The same holds true for the lack of clinical benefit of dissection of the SMA nerve plexus. The lack of a difference in either the locoregional or systemic recurrence rate in both groups could support this conclusion.

Besides lymphadenectomy, dissection of the nerve plexus has been considered an indispensable surgical technique to obtain R0 resection and subsequent reduction of locoregional recurrence. In particular, when we encounter a tumor with an extrapancreatic nerve plexus and perineural invasion such as borderline resectable cancer, or when conversion surgery for locally advanced cancer is performed, partial or half dissection of the SMA nerve plexus is definitely required to accomplish R0 resection. In this trial, the patients with invasion or suspected invasion of the SMA nerve plexus were excluded from the criteria, and we focused on the necessity of prophylactic dissection of the nerve plexus. Therefore, we believe that surgeons should strictly refrain from the prophylactic dissection of the nerve plexus because this technique can result in refractory diarrhea, malnutrition, and poor quality of life.^6^, ^7^, ^8^, ^20^

In recent years, preoperative chemotherapy or chemoradiotherapy plus adjuvant therapy in the treatment of resectable or borderline resectable pancreatic cancer have been widely spread as the multidisciplinary therapy. This trend basically indicates the difficulty to completely cure the pancreatic cancer by surgery alone. In this situation, one of the clinical benefits for this neoadjuvant therapy could be the prevention of microinvasion or micrometastasis around the primary tumor. Although our study could not demonstrate that the prophylactic dissection of the SMA right‐half nerve plexus could yield a prognostic benefit in the patients with resectable pancreatic cancer, the preoperative chemotherapy or chemoradiotherapy should be strongly recommended to reduce the incidence of local recurrence in future studies.

In conclusion, we conducted a multicenter randomized phase II trial to validate the necessity of right‐half dissection of the SMA nerve plexus in pancreatoduodenectomy for pancreatic head cancer. We did not demonstrate a clinical impact on the locoregional recurrence rate or survival outcomes. Therefore, the prophylactic dissection of the SMA nerve plexus is unnecessary given that refractory diarrhea could be induced by this technique.

## DISCLOSURE

Conflict of Interest: The authors have no commercial affiliations that can pose any conflicts of interest in connection with this study.

Funding: No funding was received for this study.

## Supporting information

Fig S1

Fig S2
