# Supplementary figures and images for: Multicenter randomized phase II trial of prophylactic right‐half dissection of superior mesenteric artery nerve plexus in pancreatoduodenectomy for pancreatic head cancer
Source: Ann Gastroenterol Surg. 2020 Sep 15;5(1):111–8. doi: 10.1002/ags3.12399 (PMC7832966; doi:10.1002/ags3.12399)

## Slide 1
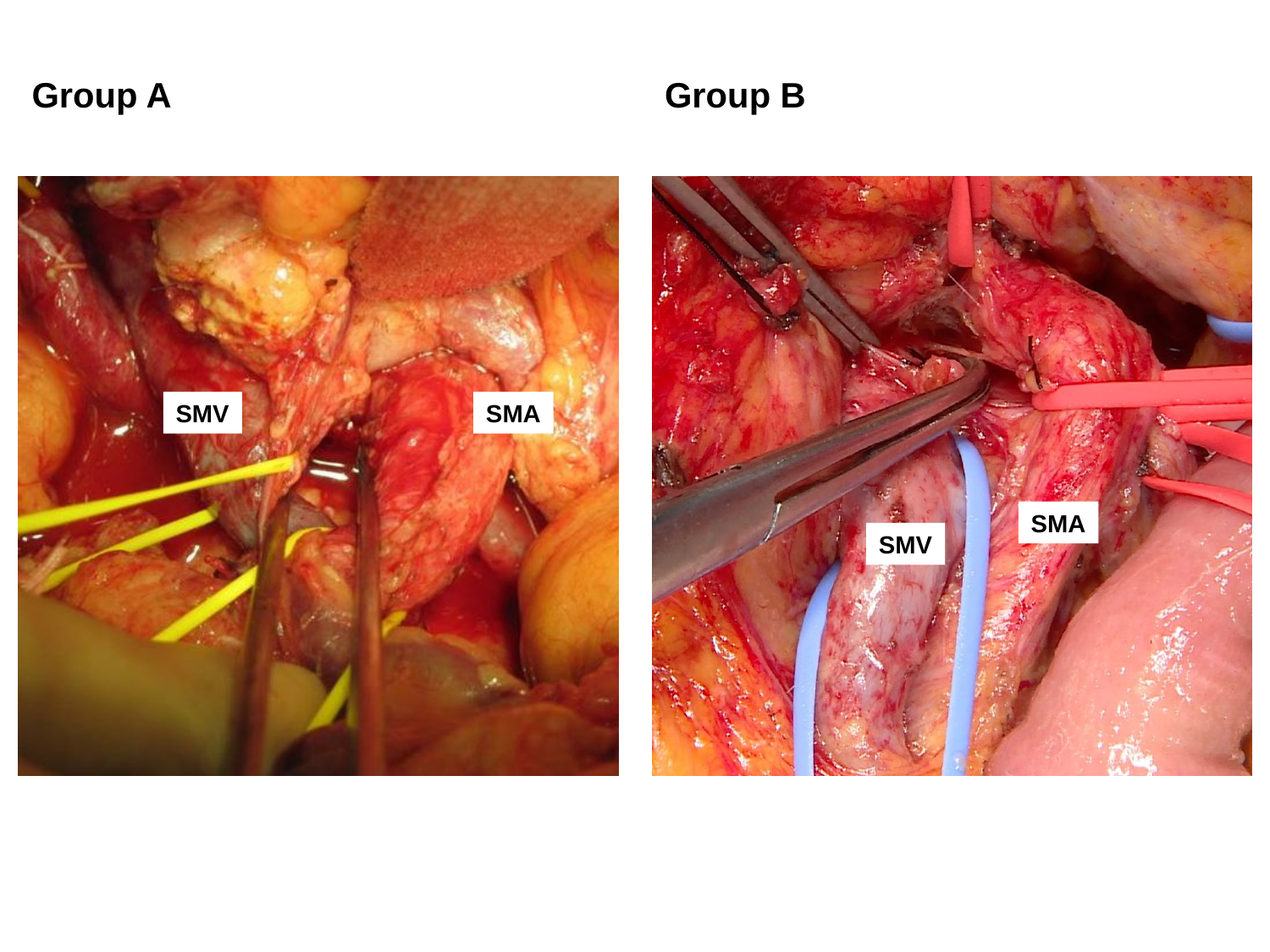

Group A
Group B
SMV
SMA
SMA
SMV

Supplement: Supplementary file 1 — Fig S1 [file AGS3-5-111-s001.pptx]

## Slide 1
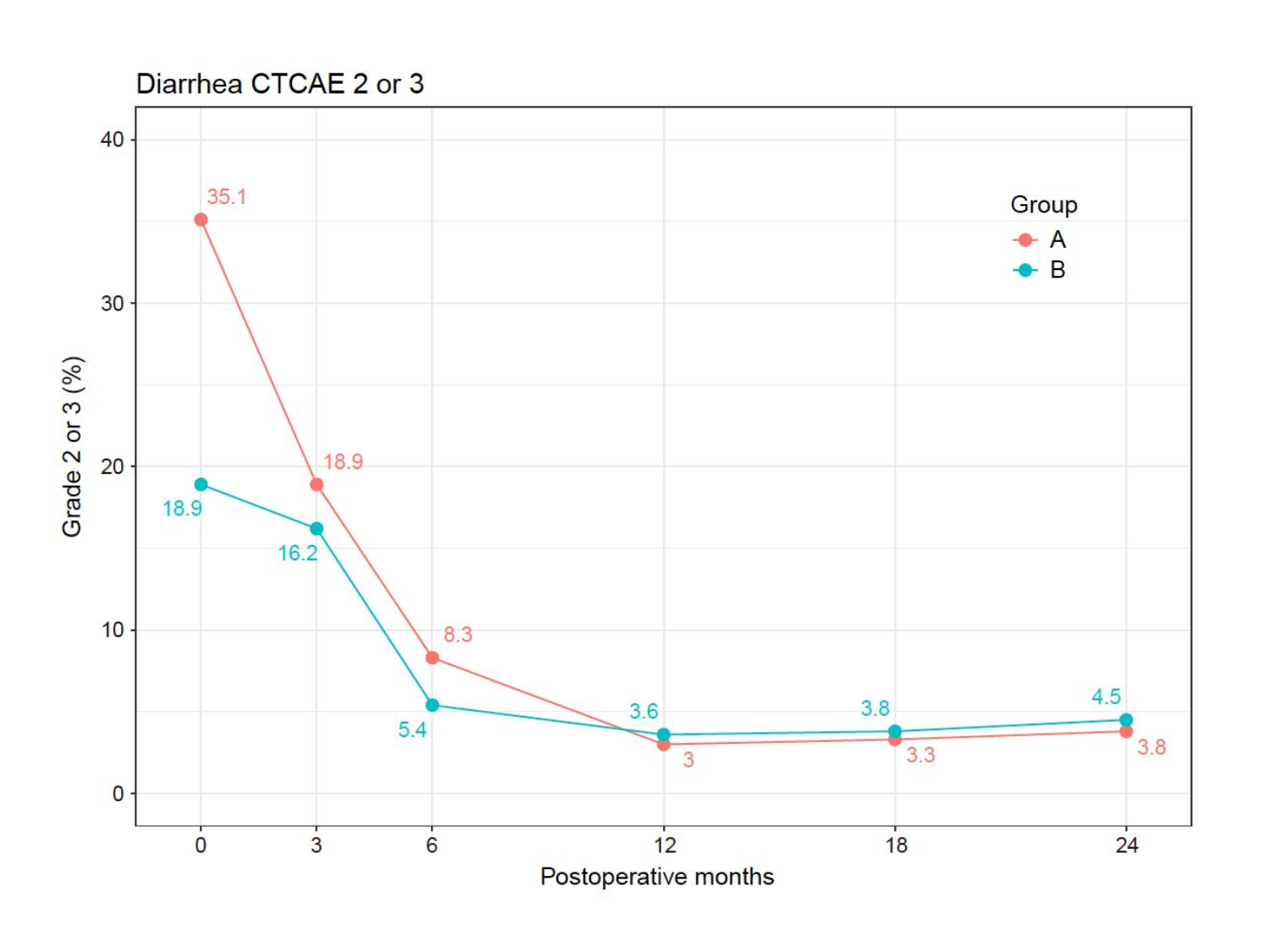

Supplement: Supplementary file 2 — Fig S2 [file AGS3-5-111-s002.pptx]
